# Can Children Discriminate Sugar-Sweetened from Non-Nutritively Sweetened Beverages and How Do They Like Them?

**DOI:** 10.1371/journal.pone.0115113

**Published:** 2014-12-31

**Authors:** Janne C. de Ruyter, Martijn B. Katan, Rosa Kas, Margreet R. Olthof

**Affiliations:** Department of Health Sciences, EMGO Institute for Health and Care Research, VU University, Amsterdam, the Netherlands; University of East Anglia, United Kingdom

## Abstract

**Background:**

Replacement of sugar-sweetened by non-nutritively sweetened beverages or water may reduce excess weight gain in children. However, it is unclear whether children like non-nutritively sweetened beverages as much as sugar-sweetened beverages. We examined whether children could taste a difference between non-nutritively sweetened beverages and matching sugar-sweetened beverages, and which of the two types of beverage they liked best.

**Methods:**

89 children aged 5 to 12 tasted seven non-nutritively sweetened beverages and matching sugar-sweetened beverages, for a total of 14 beverages. We used Triangle tests to check their ability to discriminate between the matched versions, and a 5-point scale to measure how much the children liked each individual beverage.

**Results:**

Overall, 24% of children appeared to be genuinely capable of distinguishing between non-nutritively sweetened and sugar-sweetened beverages. The mean ± SD score for how much the children liked the non-nutritively sweetened beverages was 3.39±0.7 and that for the sugar-sweetened beverages 3.39±0.6 (P = 0.9) on a scale running from 1 (disgusting) to 5 (delicious). The children preferred some beverages to others irrespective of whether they were sugar-sweetened or non-nutritively sweetened (P = 0.000). Children who correctly identified which of three drinks contained the same sweetener and which one was different also showed no preference for either type.

**Conclusion:**

We found that about one in four children were able to discriminate between non-nutritively sweetened and sugar-sweetened beverages but children liked both varieties equally. Non-nutritively sweetened beverages may therefore be an acceptable alternative to sugar-sweetened beverages although water remains the healthiest beverage for children.

## Introduction

The increased prevalence of obesity in children is a major health problem [1] that has coincided with a large increase in the consumption of sugar-sweetened beverages (SSB). Over the last several decades, the total consumption of SSB has increased worldwide and in some countries such as Mexico and the USA by almost 100% [2]. Recent large randomized controlled trials have shown that non-nutritively sweetened beverages (NNB) lead to less weight gain than SSB [3], [4]. A possible explanation is that sugars in solution are detected incompletely by receptors that determine satiation. As a result, NNB and SSB may produce similar degrees of satiety, and intake of calories from other foods is not affected [5]. Although water is by far the preferred sugar-free option for children, NNB may provide an additional alternative to SSB.

However, little is known about the ability of children to discriminate between NNB and SSB and their liking of NNB. The taste of sugar is difficult to mimic with non-nutritive sweeteners. Most non-nutritive sweeteners are perceived as bitter and as having non-sweet aftertastes [6]–[8]. Studies on beverages in adults suggest that blends of cyclamate, saccharin and acesulfame K taste more similar to sucrose than each sweetener separately [9], [10]. We are not aware of studies that examined the ability of children to discriminate between SSB and NNB.

Also, little is known about the liking of NNB in children, and results in adults are inconsistent. One study found similar ratings of pleasantness for aspartame and sucrose [11] but another study found that beverages sweetened with a blend of aspartame, acesulfame K, plus saccharin were rated less pleasant than beverages containing sucrose [12]. We are not aware of studies on preferences for NNB versus SSB in children.

We examined whether children could taste a difference between NNB and matching SSB, and which of the two varieties they liked best.

## Methods

### Ethics statement

Written informed consent was provided by a parent or guardian who had obtained assent from the child. The Medical Ethical Committee of VU University Medical Centre Amsterdam approved the study protocol. The investigation has been conducted according to the principles expressed in the Declaration of Helsinki.

### Study population

The study was done at an elementary school in the town of Purmerend near Amsterdam. In preparation for this study we recorded what drinks the children brought from home to drink during the breaks in three school classes: one with 22 children aged 4 to 6, one with 25 children aged 6 to 8, and one with 27 children aged 9 to 11. We found that 92% of the beverages consumed were sugar-sweetened (SS), and 8% were water, milk or non-nutritively sweetened (NNS). The low number of beverages sweetened with non-nutritive sweeteners agrees with findings in Dutch children in general [13]. Subsequently, we sent an information letter about the study and an informed consent form to parents of all 262 children in the school. A total of 89 (34%) children aged 5 to 12 years and their parents or guardians were willing and able to participate.

### Beverages

We used seven matched pairs of NNB and SSB, for a total of 14 beverages (Table 1). We obtained four pairs from supermarkets: Roosvicee with forest fruits and with peach flavor (H.J. Heinz Food Company Group), and Spa brand with apple/cherry and with forest fruits flavor (Spadel group). In addition, we acquired three pairs of beverages for this study from Unilever (Colworth, U.K.). These beverages were not commercially available. The flavors of these were lemon, mango, and peach. All seven NNB contained blends of sweeteners. Roosvicee peach, Spa apple/cherry, and Spa forest fruits contained cyclamate, acesulfame K plus saccharin. Non-commercial peach, mango and lemon beverages, and Roosvicee forest fruit contained sucralose plus acesulfame K.

**Table 1:** Composition of the 14 non-nutritively sweetened and sugar-sweetened beverages.

Type of beverage	Brand name	Flavour	Energy (kcal/100 ml)	Sugar (g/100 ml)	Non-nutritive sweeteners^a^
Non-nutritively sweetened	Non-commercial	Lemon	1	0.1	Sucralose, acesulfame K
	Non-commercial	Mango	1	0.1	Sucralose, acesulfame K
	Non-commercial	Peach	1	0.1	Sucralose, acesulfame K
	Roosvicee	Forest fruits	12	2.9	Sucralose, acesulfame K
	Roosvicee	Peach	14	3.5	Cyclamate, acesulfame K, saccharin
	Spa	Apple/Cherry	8	1.7	Cyclamate, acesulfame K, saccharin
	Spa	Forest fruits	8	1.7	Cyclamate, acesulfame K, saccharin
Sugar-sweetened	Non-commercial	Lemon	35	8	0
	Non-commercial	Mango	35	8	0
	Non-commercial	Peach	35	8	0
	Roosvicee	Forest fruits	39	9.6	0
	Roosvicee	Peach	40	9.9	0
	Spa	Apple/Cherry	42	10.3	0
	Spa	Forest Fruits	36	8.8	0

a The non-commercial drinks contained 0.135 g sucralose and 0.050 g acesulfame K per L. Amounts of sweeteners in the commercial drinks were not available to us.

### Procedure

We performed a single-blind sensory study in November 2008 during school hours. We administered the test in the staff room, and tested the children individually. The test lasted approximately 15 minutes per child. A total of 45 children first performed a Pleasantness test and then a Triangle test; 44 children performed these tests in reverse order. We offered 15 mL of each beverage at room temperature in a transparent 25 mL medication cup. They tasted the beverage, and then swallowed the liquid. If a child felt that one sip was not enough, she or he was allowed to take another sip. We did not ask the children to finish the whole cup. Children could drink water between beverages if they so desired.

### Sensory tests

#### Triangle test

In a Triangle test two samples are the same and one is different. The child is requested to pick out the ‘different’ sample. This test has been used previously in children [14]. We started by showing the child three shapes, two squares and one triangle, and asked him or her to point out the ‘different’ shape. We then placed 7 sets of three samples on a tray plus one set of practice samples, two identical and one different. The practice samples were commercially available beverages that were different from the study beverages. The child was asked to point out the ‘different sample’ of the practice samples. We then offered the seven sets of test beverages in the sequence described below. The child tasted the samples three at a time and pointed out which one was different from the other two.

#### Pleasantness test

This test has been validated for biscuits for children in this age group [15]. We placed 16 cups on a tray, 14 for the beverages that we were testing plus two practice samples. The practice samples were water and one SSB that was different from the study beverages. We first offered the two practice samples, and then the 14 test beverages in the sequence described below. The child pointed out the degree of pleasantness on a 5-point scale with five faces representing a range of likings from 1 =  disgusting to 5 =  delicious. For an example of the faces, see Table S3 in S1 File.

### Sequence of the beverages

For the Triangle test, all children received the beverages in the same randomly determined order [16]: Roosvicee forest fruits, Roosvicee peach, Non-commercial lemon, Non-commercial peach, Non-commercial mango, Spa forest fruits, and Spa apple/cherry. Within each of the seven sets there were six possible sequences of tasting: SAA, ASA, AAS, ASS, SAS, and SSA, where S stands for sugar-sweetened, and A for non-nutritively sweetened. These sequences were randomly allocated separately for each child to each of the seven sets. For the Pleasantness test, we generated a random sequence of the 14 beverages for each child separately [16].

### Statistical Analyses

For all data underlying the findings reported in this manuscript, see Tables S5 and S6 in S1 File. For the Triangle test, we performed one-sided binomial tests because children either could or could not identify the ‘different’ sample. For the analyses of the individual pairs, we used published tables [17]. For the analyses within groups of beverages with the same blend of non-nutritive sweeteners versus the matching SSB, and of all SSB versus all NNB beverages we pooled the data of all participants and treated them as independent observations [17]. As the number of observations now exceeded tabulated values, we converted our data to Z scores using the formula: 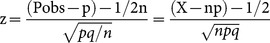
where P_obs_ is the proportion correct (X/n), X is the actual number of correct statements, n is the total number of statements, p is the chance probability of 1/3, and q = 1–p = 2/3 [18]. We then used the normal distribution tables to find the probability of obtaining this number of correct scores under the null hypothesis.

Correct answers in the Triangle test were given by children who genuinely recognized the aberrant sample, but also by children who picked out the aberrant sample by chance. We calculated the number of genuinely correct answers G as follows. Let F be the number of false answers, X is the actual number of correct statements, and n the total number. X equals n – F (Table 2). The number of correct answers X consists of genuinely correct answers plus answers correct by chance. Any subject who did not recognize the aberrant sample had a 2/3 chance of picking a false sample, which yielded F false answers, and a 1/3 chance of correctly picking out the aberrant sample, which yielded 0.5 * F answers correct by chance. Therefore the number of genuinely correct answers equals G = n–1.5 * F.

**Table 2:** Ability of children to discriminate between non-nutritively sweetened and sugar-sweetened beverages.

Beverages	Number of children	Number (%) of nominally correct responses observed^a^	Number (%) of children who accidently guessed correctly ( = false answers/2)^b^	Number (%) of children who genuinely recognized the aberrant sample^b^
Non-commercial lemon	89	43 (48)	23 (26)	20 (22)
Non-commercial mango	89	46 (52)	22 (24)	24 (27)
Non-commercial peach	89	54 (61)	18 (20)	37 (42)
Roosvicee forest fruit	89	47 (53)	21 (24)	26 (29)
Roosvicee peach	89	41 (46)	24 (27)	17 (19)
Spa apple/cherry	86	32 (37)	27 (31)	8 (9)
Spa forest fruits	88	43 (48)	23 (26)	22 (25)
All 4 beverages sweetened with sucralose/acesulfame K vs all 4 matching sugar-sweetened beverages	356	190 (53)	83 (23)	107 (30)
All 3 beverages sweetened with cyclamate/acesulfame K/saccharin vs all 3 matching sugar-sweetened beverages	263	116 (44)	74 (28)	42 (16)
All beverages combined	619	306 (49)	157 (25)	149 (24)

a The minimum number of correct responses required to reject the null hypotheses at P = 1/3 was 38 for N = 89, and 37 for N = 88 or 86.

b See 2.7 Methods section.

We analyzed the Pleasantness test with a Wilcoxon signed rank test because the data were ordinal. We analysed the results within each of the 7 drinks, within groups of drinks that contained the same blend of sweeteners, and for all NNB versus all SSB. Data were available for 89 children, except for Spa apple/cherry (N = 86) and Spa forest fruits (N = 88) where the test leader had offered the wrong samples to some children. We used both the one-way ANOVA and Kruskal-Wallis tests to analyze whether children preferred some beverages to others, irrespective of whether they were SS or NNS, including corrections for multiple testing.

For both the Triangle test and Pleasantness test, we also performed analyses for younger and older children separately. Younger children were defined as the children below the median group age of 9.3 years and older children above the median. We used the Statistical Package for the Social Sciences (SPSS) version 17.0.

## Results

### Participants

The mean age of the 89 participants was 9.2±2.0 years (±SD). 51 (57%) were girls. The school was located in an area with a z score for socio-economic status of 0.51 [19]. For comparison, an upper class neighborhood in Amsterdam had a z score of 2.97. The mean score of all Dutch neighborhoods is zero by definition.

### Triangle test

The percentage of children who correctly pointed out the ‘different’ sample ranged from 37% to 61% between the seven beverages (Table 2). When results for all SSB versus all NNB were added up, 306 of the 619 responses or 49% were correct (P<0.001). After correction for chance answers (See 2.7 Methods section) 24% of children were truly competent to distinguish between NNB and SSB. Results were similar when we pooled drinks by blend of sweeteners. Out of 356 responses, 190 or 53% correctly distinguished drinks sweetened with sucralose plus acesulfame K from matching drinks sweetened with sugar. After correction for chance answers this leaves 107 genuinely correct answers, suggesting that 30% of children genuinely distinguished the sucralose plus acesulfame K blend from drinks sweetened with sugar (P<0.001). For the cyclamate, acesulfame K plus saccharin blend, 116 of the 263 responses or 44% were nominally correct (P<0.001). Correction for chance suggests that 16% of children could genuinely distinguish this blend of sweeteners from sugar. Younger and older children performed similarly on the Triangle test, see Table S1 in S1 File. The different sample was identified correctly in 165 or 54% of the 307 Triangle tests that were administered of the younger children. In the older children this was true for 149 or 48% of the 312 Triangle tests. Younger children seemed thus even slightly better at discriminating between the two. However, the difference between younger and older children was not statistically significant (P = 0.14).

### Pleasantness test

For each of the seven pairs separately, children liked the SS and the NNS beverages similarly, Table 3 (for the frequencies of scores on the five point scale for each drink, see Table S3 in S1 File). When we compared all NNB with all SSB, children liked both types equally, with mean scores of 3.4 for both (P = 0.90). We found similar results when we pooled drinks by blend of sweeteners and compared them with their matching SSB (Table 3). Liking of SSB equaled liking of the corresponding NNB both for children who could and for those who could not point out the ‘different’ sample correctly in the Triangle test (‘correct tasters’ P = 0.46, ‘false tasters’ P = 0.48). We also found that both younger and older children liked SSB and NNB similarly, see Table S2 in S1 File. The younger children showed a mean ± SD liking of 3.6±0.54 for all SSB, and of 3.5±0.73 for all NNB. The older children showed a mean ± SD liking of 3.2±0.63 for all SSB, and of 3.2±0.59 for all NNB. The differences in liking of SSB vs NNB were not significantly different for both younger and older children (P>0.05).

**Table 3:** Rating of pleasantness of non-nutritively sweetened and sugar-sweetened beverages by 89 children^a^.

Beverage	Non-nutritively sweetened	Sugar-sweetened	P for difference
	Mean (SD)	Mean (SD)	
Non-commercial lemon	3.7±1.1^b^	3.7±1.1	0.73
Non-commercial mango	3.2±1.1	3.2±1.1	0.84
Non-commercial peach	3.3±1.2	3.1±1.2	0.48
Roosvicee forest fruits	3.2±1.3	3.1±1.3	0.41
Roosvicee peach	3.2±1.2	3.2±1.2	0.64
Spa apple/cherry	3.5±1.2	3.6±1.0	0.33
Spa forest fruits	3.7±1.2	3.8±1.0	0.35
All 4 beverages sweetened with sucralose/acesulfame K vs all 4 matching sugar-sweetened beverages	3.3±0.8	3.3±0.8	0.41
All 3 beverages sweetened with cyclamate/acesulfame K/saccharin vs all 3 matching sugar-sweetened beverages	3.5±0.8	3.5±0.7	0.42
All beverages combined	3.4±0.7	3.4±0.6	0.90

a Pleasantness was rated on a 5-point scale of liking from 1 =  disgusting to 5 =  delicious [5].

b The median was 3.0 for all beverages except for non-commercial lemon (both non-nutritively sweetened and sugar-sweetened), Spa apple/cherry and Spa forest fruits (both non-nutritively sweetened and sugar-sweetened) that were scored with a median of 4.

The children preferred some beverages to others irrespective of whether they were SS or NNS (P = 0.000 for both one-way ANOVA and Kruskal-Wallis analyses). For instance, we found that children liked the SS Spa forest fruits significantly better than both the SS and NNS variety of non-commercial mango, non-commercial peach, Roosvicee forest fruit, Roosvicee peach drink, see Table S4a and Table S4b in S1 File. Similarly, as depicted in Table 3, the mean ± SD liking of the SS Spa forest fruits was rated 3.8±1.0, and thus significantly higher than SS non –commercial mango 3.2±1.1, NNS non-commercial mango 3.2±1.1, SS non –commercial peach 3.1±1.2, NNS non-commercial peach 3.3±1.2, SS Roosvicee forest fruit 3.1±1.3, NNS Roosvicee forest fruit 3.2±1.3, SS Roosvicee peach 3.2±1.2, and NNS Roosvicee peach 3.2±1.2.

## Discussion

We found that about one in four children was genuinely able to discriminate between SSB and NNB when they were asked to identify the odd sample out of three. Evidently the blends of non-nutritive sweeteners used in our beverages tasted somewhat different from sugar, but the difference was slight [9], [10]. Children preferred some beverages to others irrespective of whether they were SS or NNS.

Interestingly, the beverages were liked equally, even by the children who were able to discriminate between SSB and NNB. This result is in line with a previous three-week study in adults with beverages [11], and with studies of other sweetened foods such as pudding [20] or cream cheese [21] which also found that SS and NNS products were liked equally. It is also in line with previous studies that showed that adjustments for flavor did not yield different outcomes [22]. In contrast, beverages sweetened with a blend of aspartame, acesulfame K, plus saccharin were rated lower in pleasantness than beverages containing sugar when large amounts were consumed one day per week [12]. The cause for this discrepancy is unclear.

We calculated that only some 24% of children was genuinely competent to distinguish between non-nutritive sweeteners and sugar. We would not expect a higher proportion in other populations, because at the school where our study was done the large majority of children habitually consumed SSB and very few drank NNB during school breaks (See 2.2 Methods section). The taste of NNB was therefore foreign to them. Adults who drink mostly SSB are better able to recognize NNB than habitual consumers of NNB [23]. Our study suggests that this may also apply to children. We also found that children were better at discriminating beverages sweetened with sucralose/acesulfame K blend from their sugar-sweetened counterparts than the beverages with the cyclamate/acesulfame K/saccharin blend. We therefore speculate that the resemblance in liking of non-nutritively sweetened products to their sugar-sweetened counterparts may highly depend on the type and mix of sweeteners used.

Our seven NNB still contained small amounts of sugar. Small amounts of sugar are sometimes added to NNB to improve the taste. However, our data do not support such an effect. The three NNB with 0.1% of sugar were liked as much as the four others that contained 1.7% to 3.5% sugar. Apparently, residual sugar content is not a major determinant of the taste of this type of NNB in children.

Our study has several strengths. To our best knowledge, this was the first sensory study of non-nutritive sweeteners in children. We included 89 subjects while other studies had only 16 to 31 participants [11], [20], [21]. We also generated random sequences of the beverages for each child separately, which removed the bias that may occur when the sequence of tasting affects the judgments of products [24]. Finally, we used non-carbonated beverages that facilitate the perception of taste [10]. Our study also has limitations. It was limited to non-carbonated fruit-flavored drinks and our results may not hold for types of drinks, e.g. carbonated drinks. Also, we do not know the exact amounts of non-nutritive sweeteners in the NNB obtained from supermarkets, but the amounts were evidently such that the children liked these drinks as much as their sugar-sweetened counterparts.

Both the one-way ANOVA and Kruskal-Wallis analyses showed that children preferred some beverages to others irrespective of whether they were SS or NNS. On one hand the Kruskal-Wallis test is more reliable here because it takes into account the ordinal structure of the data and the lack of normal distributions in our outcome measures. However, a disadvantage of this test is that it uses the median instead of the mean and is therefore not able to pick up more subtle differences in liking between drinks. We therefore present the outcomes of both analyses, see Table S4a and S4b in S1 File.

Our participants were healthy Dutch children. Future studies should be carried out to investigate whether our findings hold for other ethnic groups, obese children, habitual consumers of NNB, and different age groups.

In conclusion, although one in four children were able to discriminate between NNB and SSB, they liked both types equally. Recent large randomized controlled trials have shown that replacement of SSB by NNB reduces weight gain in children and adolescents [3], [4]. Therefore NNB may provide a useful alternative to SSB. However, water is the most preferred option, since all sweetened drinks and juices cause dental erosion which is a major health threat in children [25].

## Supporting Information

S1 File **Supplementary Appendix.** **Table S1**, Ability of children to discriminate between non-nutritively sweetened and sugar-sweetened beverages by all participants, older and younger children. **Table S2**, Rating of pleasantness of non-nutritively sweetened and sugar-sweetened beverages by all participants, older and younger children. **Table S3**, Frequencies of scores on the five point scale for each drink on the Pleasantness test by 89 children. **Table S4**, A)The extent to which the 89 children rated one beverage better than the other irrespective of the sweetener used analyzed with one-way ANOVA analyses. B) The extent to which the 89 children rated one beverage better than the other irrespective of the sweetener used analyzed with Kruskal-Wallis tests. **Table S5**, The full dataset of both the Triangle and Pleasantness test by 89 children. **Table S6**, The full dataset of both the Triangle and Pleasantness test by 89 children.
(DOCX)
