# Supplementary material for: Can Children Discriminate Sugar-Sweetened from Non-Nutritively Sweetened Beverages and How Do They Like Them?
Source: PLoS One. 2014 Dec 31;9(12):e115113. doi: 10.1371/journal.pone.0115113 (PMC4281215; doi:10.1371/journal.pone.0115113)
Supplement: S1 File — Supplementary Appendix. Table S1, Ability of children to discriminate between non-nutritively sweetened and sugar-sweetened beverages by all participants, older and younger children. Table S2, Rating of pleasantness of non-nutritively sweetened and sugar-sweetened beverages by all participants, older and younger children. Table S3, Frequencies of scores on the five point scale for each drink on the Pleasantness test by 89 children. Table S4, A)The extent to which the 89 children rated one beverage better than the other irrespective of the sweetener used analyzed with one-way ANOVA analyses. B) The extent to which the 89 children rated one beverage better than the other irrespective of the sweetener used analyzed with Kruskal-Wallis tests. Table S5, The full dataset of both the Triangle and Pleasantness test by 89 children. Table S6, The full dataset of both the Triangle and Pleasantness test by 89 children. (DOCX) [file pone.0115113.s001.docx]

**File S1: Supplementary Appendix**

Supplement to: Janne C de Ruyter, Martijn B Katan, Rosa Kas, Margreet R Olthof . Can children discriminate sugar-sweetened from non- nutritively sweetened beverages and how do they like them?

Page 2: Table S1 in File S1: Ability of children to discriminate between non-nutritively sweetened and sugar-sweetened beverages by all participants, older and younger children.

Page 3: Table S2 in File S1: Rating of pleasantness of non-nutritively sweetened and sugar-sweetened beverages by all participants, older and younger children.

Page 4: Table S3 in File S1: Frequencies of scores on the five point scale for each drink on the Pleasantness test by 89 children.

Page 6: Table S4a in File S1: The extent to which the 89 children rated one beverage better than the other irrespective of the sweetener used analyzed with one-way ANOVA analyses.

Page 8: Table S4b in File S1: The extent to which the 89 children rated one beverage better than the other irrespective of the sweetener used analyzed with Kruskal-Wallis tests.

Page 10: Table S5 and Table S6 in File S1: The full dataset of both the Triangle and Pleasantness test by 89 children.

| **Table S1. Ability of children to discriminate between non-nutritively sweetened and sugar-sweetened beverages by all participants, older and younger children.** | | | | | | |
| --- | --- | --- | --- | --- | --- | --- |
| Beverages | Number of children | Number (%) of nominally correct responses observed | Number of younger  children (<= 9.3 y) a | Number (%) of nominally correct responses observed | Number of older children (> 9.3 y) | Number (%) of nominally correct responses observed |
| Non-commercial lemon | 89 | 43 (48) | 44 | 20 (46) | 45 | 23 (51) |
| Non-commercial mango | 89 | 46 (52) | 44 | 25 (57) | 45 | 21 (47) |
| Non-commercial peach | 89 | 54 (61) | 44 | 24 (55) | 45 | 30 (67) |
| Roosvicee forest fruit | 89 | 47 (53) | 44 | 24 (55) | 45 | 23 (51) |
| Roosvicee peach | 89 | 41 (46) | 44 | 21 (48) | 45 | 20 (44) |
| Spa apple/cherry | 86 | 32 (37) | 44 | 15 (34) | 45 | 17 (38) |
| Spa forest fruits | 88 | 43 (48) | 43 | 17 (39) | 45 | 26 (58) |
| a We divided the participants in the oldest and youngest 50% based on the median of 9.3 years old . | | | | | | |

| **Table S2. Rating of pleasantness of non-nutritively sweetened and sugar-sweetened beverages by all participants, older and younger children.** | | | | | | |
| --- | --- | --- | --- | --- | --- | --- |
| Beverages | All participants  (N=89) | All participants  (N=89) | Younger children (N=44)a | Younger children (N=44) | Older children (N=45) | Older Children (N=45) |
|  | NNSb  Mean±SD | SS Mean±SD | NNS Mean±SD | SS Mean±SD | NNS Mea±SD | SS Mean±SD |
| Non-commercial lemon | 3.7±1.1 | 3.7±1.1 | 3.9±1.1 | 4.0±1.1 | 3.5±1.1 | 3.5±1.1 |
| Non-commercial mango | 3.2±1.1 | 3.2±1.1 | 3.3±1.3 | 3.4±1.2 | 3.0±0.8 | 3.0±1.0 |
| Non-commercial peach | 3.3±1.2 | 3.1±1.2 | 3.6±1.2 | 3.3±1.1 | 3.0±1.2 | 2.9±1.2 |
| Roosvicee forest fruit | 3.2±1.3 | 3.1±1.3 | 3.3±1.3 | 3.3±1.2 | 3.2±1.2 | 2.9±1.3 |
| Roosvicee peach | 3.2±1.2 | 3.2±1.2 | 3.5±1.3 | 3.2±1.3 | 3.0±1.1 | 3.1±1.1 |
| Spa apple/cherry | 3.5±1.2 | 3.6±1.0 | 3.5±1.1 | 3.9±1.0 | 3.5±1.2 | 3.4±1.1 |
| Spa forest fruits | 3.7±1.2 | 3.8±1.0 | 3.7±1.2 | 3.9±1.0 | 3.6±1.3 | 3.7±1.0 |
| a We divided the participants in the oldest and youngest 50% based on the median of 9.3 years old; younger are children <= 9.3 years, older children > 9.3 years.  b NNS refers to ‘non-nutritively sweetened’, SS refers ‘sugar-sweetened’ | | | | | | |


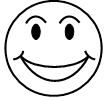

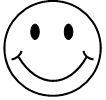

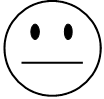

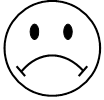

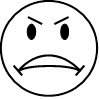


| **Table S3. Frequencies of scores on the five point scale for each drink on the Pleasantness test by 89 children** | | | | | | | |
| --- | --- | --- | --- | --- | --- | --- | --- |
|  |  | Ik vind dit drankje **heel erg lekker *(****I find this drink* ***delicious)*** a | Ik vind dit drankje l**ekker** *(I* ***like*** *this drink)* | Ik vind dit drankje **niet vies maar ook niet lekker**  *(I* ***neither like***  ***nor dislike***  *this drink)* | Ik vind dit drankje **een beetje vies** *(I* ***do not like*** *this drink)* | Ik vind dit drankje **heel erg vies**  *(I find this drink*  ***disgusting****)* | Total number of  responses |
| Non- nutritively sweetened | Non- commercial lemon | 4 | 10 | 21 | 28 | 26 | 89 |
|  | Non- commercial mango | 9 | 9 | 40 | 19 | 12 | 89 |
|  | Non- commercial peach | 8 | 18 | 23 | 23 | 17 | 89 |
|  | Roosvicee  forest fruits | 12 | 12 | 26 | 24 | 15 | 89 |
|  | Roosvicee  peach | 7 | 21 | 21 | 24 | 16 | 89 |
|  | Spa  apple/cherry | 6 | 10 | 26 | 28 | 19 | 89 |

|  | Spa forest fruits | 8 | 8 | 17 | 30 | 26 |  |
| --- | --- | --- | --- | --- | --- | --- | --- |
| Sugar- sweetened | Non- commercial lemon | 3 | 10 | 24 | 22 | 30 | 89 |
|  | Non- commercial mango | 5 | 17 | 35 | 19 | 13 | 89 |
|  | Non- commercial peach | 10 | 13 | 33 | 22 | 11 | 89 |
|  | Roosvicee  forest fruits | 14 | 12 | 21 | 23 | 12 | 89 |
|  | Roosvicee  peach | 8 | 21 | 23 | 24 | 13 | 89 |
|  | Spa  apple/cherry | 3 | 10 | 23 | 35 | 18 | 89 |
|  | Spa forest fruits | 2 | 5 | 28 | 28 | 26 | 89 |
| a We added the English translation in *italics* | | | | | | |  |

| **Table S4a. The extent to which the 89 children rated one beverage better than the other irrespective of the sweetener used analyzed with one-way ANOVA analyses.** | | | | | | | | | | | | | | | | | |
| --- | --- | --- | --- | --- | --- | --- | --- | --- | --- | --- | --- | --- | --- | --- | --- | --- | --- |
|  |  | **Non-nutritively sweetened** | | | | | | | | | **Sugar-sweetened** | | | | | | |
|  |  | Non- commercial lemon | Non- commercial mango | Non- commercial peach | Roos-  vicee  forest fruits | Roos-  vicee  peach | Spa  apple/  cherry | Spa  forest fruits | | | Non- commercial lemon | Non- commercial mango | Non- commercial peach | Roos-  vicee  forest  fruits | Roos-  vicee  peach | Spa  apple/  cherry | Spa  forest fruits |
| **Non-nutritively sweetened** | Non- commercial lemon |  | **+ a** | **+** | **+** | **+** |  | |  |  | | **+** | **+** | **+** | **+** |  |  |
|  | Non- commercial mango | **-** |  |  |  |  | **-** | | **-** | **-** | |  |  |  |  | **-** | **-** |
|  | Non- commercial peach | **-** |  |  |  |  |  | | **-** | **-** | |  |  |  |  | **-** | **-** |
|  | Roosvicee  forest fruit | **-** |  |  |  |  |  | | **-** | **-** | |  |  |  |  | **-** | **-** |
|  | Roosvicee  peach | **-** |  |  |  |  |  | | **-** | **-** | |  |  |  |  | **-** | **-** |
|  | Spa  apple/  cherry |  | **+** |  |  |  |  | |  |  | |  | **+** | **+** | **+** |  |  |
|  | Spa  forest fruits |  | **+** | **+** | **+** | **+** |  | |  |  | | **+** | **+** | **+** | **+** |  |  |
|  | Non- commercial lemon |  | **+** | **+** | **+** | **+** |  | |  |  | | **+** | **+** | **+** | **+** |  |  |

| **Sugar-sweetened** | Non- commercial mango | **-** |  |  |  |  |  | **-** | **-** |  |  |  |  | **-** | **-** |
| --- | --- | --- | --- | --- | --- | --- | --- | --- | --- | --- | --- | --- | --- | --- | --- |
|  | Non- commercial peach | **-** |  |  |  |  | **-** | **-** | **-** |  |  |  |  | **-** | **-** |
|  | Roosvicee  forest fruits | **-** |  |  |  |  | **-** | **-** | **-** |  |  |  |  | **-** | **-** |
|  | Roosvicee  peach | **-** |  |  |  |  | **-** | **-** | **-** |  |  |  |  | **-** | **-** |
|  | Spa  apple/  cherry |  | **+** | **+** | **+** | **+** |  |  |  | **+** | **+** | **+** | **+** |  |  |
|  | Spa  forest fruits |  | **+** | **+** | **+** | **+** |  |  |  | **+** | **+** | **+** | **+** |  |  |
| a A ‘+ sign’ in the cell means that the drink on the x-axis is liked significantly better than the drink on the y-axis, whereas a ‘- sign’ means that the drink on the x-axis is liked significantly less than the drink on the y-axis. For example, sugar-sweetened Spa forest fruits is liked significantly better than sugar-sweetened and non-nutritively sweetened non-commercial mango, non-commercial peach, Roosvicee forest fruits and Roosvicee peach. An empty cell means that there was no significant association. Differences in liking between the beverages were analyzed with the use of one-way ANOVA; P≤ 0.05 was considered to indicate significance. | | | | | | | | | | | | | | | |

| **Table S4b. The extent to which the 89 children rated one beverage better than the other irrespective of the sweetener used analyzed with Kruskal-Wallis tests.** | | | | | | | | | | | | | | | | | |
| --- | --- | --- | --- | --- | --- | --- | --- | --- | --- | --- | --- | --- | --- | --- | --- | --- | --- |
|  |  | **Non-nutritively sweetened** | | | | | | | | | **Sugar-sweetened** | | | | | | |
|  |  | Non- commercial lemon | Non- commercial mango | Non- commercial peach | Roos-  vicee  forest fruits | Roos-  vicee  peach | Spa  apple/  cherry | Spa  forest fruits | | | Non- commercial lemon | Non- commercial mango | Non- commercial peach | Roos-  Vice  forest  fruits | Roos-  vicee  peach | Spa  apple/  cherry | Spa  forest fruits |
| **Non-nutritively sweetened** | Non- commercial lemon |  |  |  |  |  |  | |  |  | |  |  |  |  |  |  |
|  | Non- commercial mango | **- a** |  |  |  |  |  | | **-** |  | |  |  |  |  | **-** | **-** |
|  | Non- commercial peach |  |  |  |  |  |  | |  |  | |  |  |  |  |  |  |
|  | Roosvicee  forest fruit |  |  |  |  |  |  | |  |  | |  |  |  |  |  |  |
|  | Roosvicee  peach |  |  |  |  |  |  | |  |  | |  |  |  |  |  |  |
|  | Spa  apple/  cherry |  |  |  |  |  |  | |  |  | |  |  |  |  |  |  |
|  | Spa  forest fruits |  |  |  |  |  |  | |  |  | |  |  |  |  |  |  |
|  | Non- commercial lemon |  |  |  |  |  |  | |  |  | |  |  |  |  |  |  |

| **Sugar-sweetened** | Non- commercial mango | **-** |  |  |  |  |  | **-** |  |  |  |  |  |  | **-** |
| --- | --- | --- | --- | --- | --- | --- | --- | --- | --- | --- | --- | --- | --- | --- | --- |
|  | Non- commercial peach |  |  |  |  |  |  | **-** |  |  |  |  |  |  |  |
|  | Roosvicee  forest fruits |  |  |  |  |  |  |  |  |  |  |  |  |  |  |
|  | Roosvicee  peach |  |  |  |  |  |  |  |  |  |  |  |  |  |  |
|  | Spa  apple/  cherry |  |  |  |  |  |  |  |  |  |  |  |  |  |  |
|  | Spa  forest fruits |  |  |  |  |  |  |  |  |  |  |  |  |  |  |
| a A ‘+ sign’ in the cell means that the drink on the x-axis is liked significantly better than the drink on the y-axis, whereas a ‘- sign’ means that the drink on the x-axis is liked significantly less than the drink on the y-axis. For example, non-nutritively sweetened non-commercial mango is liked significantly less than non-nutritively sweetened non-commercial lemon, non-nutritively sweetened Spa forest fruits, sugar-sweetened Spa forest fruits, and sugar-sweetened Spa apple/cherry. An empty cell means that there was no significant association. Differences in liking between the beverages were analyzed with the use of Kruskal-Wallis Tests; P≤ 0.10 was considered to indicate significance. | | | | | | | | | | | | | | | |

**Table S5 and S6. The full dataset of both the Triangle and Pleasantness test by 89 children**

| **Table S5. Ability of children to discriminate between non‐nutritively sweetened and sugar‐**  **sweetened beverages. 0 refers to a false, and 1 to a correct answer** | | | | | | | | |
| --- | --- | --- | --- | --- | --- | --- | --- | --- |
| Participant number | Age (y) | Roosvicee  forest fruits | Roosvicee peach | Non‐  commercial lemon | Non‐  commercial peach | Non‐  commercial mango | Spa  forest fruits | Spa  apple/  cherry |
| 1 | 11,81 | 0 | 1 | 0 | 1 | 0 | Missing | Missing |
| 2 | 10,28 | 1 | 0 | 1 | 0 | 0 | 0 | Missing |
| 3 | 8,79 | 1 | 1 | 1 | 1 | 1 | 0 | Missing |
| 4 | 5,54 | 1 | 0 | 0 | 0 | 0 | 0 | 0 |
| 5 | 11,96 | 0 | 1 | 1 | 1 | 1 | 1 | 1 |
| 6 | 11,24 | 1 | 1 | 1 | 1 | 1 | 0 | 0 |
| 7 | 12,28 | 1 | 0 | 1 | 1 | 1 | 1 | 1 |
| 8 | 10,35 | 0 | 1 | 1 | 1 | 0 | 1 | 1 |
| 9 | 10,9 | 0 | 1 | 0 | 1 | 1 | 1 | 1 |
| 10 | 10,13 | 0 | 1 | 0 | 1 | 0 | 1 | 0 |
| 11 | 9,88 | 1 | 0 | 0 | 1 | 1 | 0 | 0 |
| 12 | 9,33 | 0 | 1 | 0 | 0 | 0 | 0 | 1 |
| 13 | 11,09 | 0 | 1 | 0 | 0 | 0 | 1 | 0 |
| 14 | 10,14 | 1 | 0 | 0 | 1 | 1 | 1 | 0 |
| 15 | 9,61 | 0 | 0 | 1 | 1 | 0 | 1 | 1 |
| 16 | 11,17 | 1 | 0 | 1 | 1 | 1 | 1 | 0 |
| 17 | 10,51 | 0 | 1 | 0 | 0 | 0 | 0 | 0 |
| 18 | 10,21 | 1 | 0 | 1 | 1 | 1 | 1 | 1 |
| 19 | 9,19 | 1 | 1 | 1 | 1 | 1 | 0 | 0 |
| 20 | 11,38 | 0 | 0 | 1 | 1 | 0 | 1 | 1 |
| 21 | 9,7 | 0 | 1 | 0 | 0 | 1 | 1 | 0 |
| 22 | 10,25 | 0 | 1 | 0 | 1 | 0 | 0 | 0 |
| 23 | 12,13 | 0 | 0 | 0 | 0 | 1 | 1 | 0 |
| 24 | 9,69 | 0 | 0 | 0 | 1 | 0 | 0 | 0 |
| 25 | 11,91 | 0 | 0 | 0 | 1 | 0 | 1 | 0 |
| 26 | 11,72 | 1 | 1 | 1 | 0 | 0 | 0 | 0 |
| 27 | 11,78 | 1 | 1 | 1 | 0 | 0 | 0 | 1 |
| 28 | 7,3 | 1 | 0 | 0 | 1 | 1 | 1 | 0 |
| 29 | 7,36 | 0 | 1 | 1 | 1 | 1 | 0 | 0 |
| 30 | 7,3 | 1 | 1 | 0 | 1 | 1 | 0 | 1 |
| 31 | 8,66 | 1 | 0 | 1 | 0 | 0 | 0 | 0 |
| 32 | 8,51 | 1 | 1 | 0 | 0 | 0 | 0 | 1 |
| 33 | 8,32 | 0 | 1 | 1 | 1 | 0 | 1 | 0 |
| 34 | 8,56 | 1 | 0 | 1 | 1 | 0 | 0 | 1 |
| 35 | 8,49 | 1 | 0 | 1 | 1 | 1 | 1 | 1 |
| 36 | 9,33 | 0 | 0 | 1 | 0 | 1 | 0 | 1 |
| 37 | 8,41 | 0 | 0 | 0 | 0 | 1 | 1 | 1 |
| 38 | 8,78 | 0 | 1 | 0 | 0 | 1 | 0 | 0 |
| 39 | 8,83 | 1 | 1 | 0 | 0 | 1 | 0 | 0 |
| 40 | 11,57 | 1 | 0 | 0 | 1 | 1 | 1 | 0 |
| 41 | 11,07 | 1 | 0 | 0 | 0 | 0 | 0 | 0 |
| 42 | 10,93 | 0 | 1 | 1 | 1 | 0 | 1 | 1 |

| 43 | 10,2 | 1 | 0 | 0 | 0 | 0 | 0 | 0 |
| --- | --- | --- | --- | --- | --- | --- | --- | --- |
| 44 | 10,24 | 0 | 1 | 1 | 1 | 0 | 1 | 1 |
| 45 | 10,35 | 0 | 0 | 1 | 0 | 1 | 0 | 1 |
| 46 | 9,67 | 1 | 0 | 0 | 1 | 1 | 1 | 0 |
| 47 | 9,56 | 0 | 0 | 0 | 1 | 0 | 1 | 1 |
| 48 | 11,32 | 1 | 0 | 1 | 1 | 0 | 1 | 0 |
| 49 | 11,74 | 0 | 0 | 1 | 1 | 1 | 1 | 0 |
| 50 | 11,32 | 1 | 1 | 1 | 1 | 1 | 1 | 1 |
| 51 | 11,85 | 1 | 1 | 1 | 0 | 1 | 0 | 1 |
| 52 | 10,5 | 1 | 0 | 0 | 0 | 0 | 0 | 1 |
| 53 | 10,44 | 1 | 1 | 0 | 0 | 1 | 0 | 0 |
| 54 | 10,93 | 1 | 0 | 0 | 1 | 1 | 1 | 0 |
| 55 | 11,24 | 1 | 0 | 1 | 1 | 0 | 0 | 0 |
| 56 | 11,36 | 1 | 0 | 0 | 1 | 1 | 1 | 0 |
| 57 | 9,9 | 0 | 1 | 1 | 1 | 0 | 1 | 1 |
| 58 | 9,7 | 1 | 1 | 0 | 1 | 1 | 0 | 0 |
| 59 | 9,78 | 1 | 1 | 1 | 0 | 0 | 0 | 0 |
| 60 | 8,65 | 0 | 1 | 0 | 0 | 0 | 1 | 1 |
| 61 | 7,33 | 1 | 0 | 1 | 0 | 1 | 1 | 1 |
| 62 | 9,05 | 1 | 0 | 1 | 0 | 0 | 0 | 1 |
| 63 | 8,08 | 1 | 1 | 1 | 0 | 0 | 0 | 0 |
| 64 | 8,17 | 1 | 1 | 0 | 1 | 1 | 0 | 0 |
| 65 | 7,83 | 1 | 0 | 1 | 0 | 1 | 0 | 0 |
| 66 | 6,4 | 0 | 0 | 0 | 1 | 0 | 1 | 0 |
| 67 | 7,17 | 0 | 0 | 0 | 1 | 1 | 1 | 1 |
| 68 | 6,87 | 0 | 1 | 0 | 0 | 1 | 0 | 0 |
| 69 | 11,46 | 0 | 0 | 1 | 1 | 0 | 1 | 0 |
| 70 | 5,16 | 1 | 1 | 0 | 1 | 0 | 0 | 0 |
| 71 | 5,86 | 0 | 1 | 0 | 1 | 1 | 0 | 0 |
| 72 | 6,52 | 0 | 0 | 1 | 0 | 0 | 1 | 1 |
| 73 | 5,37 | 0 | 0 | 0 | 0 | 1 | 0 | 0 |
| 74 | 5,67 | 1 | 0 | 1 | 0 | 0 | 0 | 0 |
| 75 | 5,99 | 0 | 0 | 0 | 1 | 1 | 1 | 0 |
| 76 | 6,01 | 1 | 0 | 1 | 1 | 1 | 0 | 0 |
| 77 | 5,28 | 0 | 1 | 1 | 1 | 0 | 0 | 0 |
| 78 | 5,78 | 1 | 1 | 0 | 0 | 1 | 0 | 1 |
| 79 | 5,61 | 0 | 0 | 0 | 1 | 0 | 0 | 0 |
| 80 | 5,83 | 0 | 0 | 1 | 1 | 0 | 0 | 0 |
| 81 | 8,47 | 1 | 0 | 0 | 0 | 1 | 1 | 0 |
| 82 | 8,11 | 0 | 0 | 1 | 1 | 1 | 1 | 0 |
| 83 | 8,59 | 1 | 0 | 0 | 0 | 0 | 0 | 1 |
| 84 | 8,72 | 1 | 1 | 1 | 1 | 1 | 1 | 1 |
| 85 | 7,85 | 1 | 0 | 0 | 1 | 1 | 1 | 0 |
| 86 | 8,7 | 1 | 1 | 1 | 1 | 0 | 1 | 0 |
| 87 | 9,06 | 0 | 1 | 0 | 0 | 1 | 1 | 0 |
| 88 | 7,29 | 0 | 1 | 0 | 1 | 1 | 0 | 1 |
| 89 | 8,14 | 0 | 0 | 1 | 1 | 0 | 1 | 0 |

| **Table S6. Rating of pleasantness of non‐nutritively sweetened and sugar‐sweetened beverages by 89 children. Pleasantness was rated** | | | | | | | | | |
| --- | --- | --- | --- | --- | --- | --- | --- | --- | --- |
|  | Sugar‐  sweetened | Non‐ nutritively sweetened | Sugar‐  sweetened | Non‐ nutritively sweetened | Sugar‐  sweetened | Non‐ nutritively sweetened | Sugar‐  sweetened | Non‐ nutritively sweetened | Sugar‐  sweetened |
| Participant number | Roosvicee peach | Roosvicee peach | Roosvicee forest fruits | Roosvicee forest fruits | Non‐ commercial lemon | Non‐ commercial lemon | Non‐ commercial peach | Non‐ commercial peach | Non‐ commercial mango |
| 1 | 2 | 3 | 1 | 4 | 5 | 5 | 2 | 2 | 3 |
| 2 | 2 | 3 | 1 | 4 | 4 | 5 | 5 | 5 | 4 |
| 3 | 2 | 1 | 4 | 4 | 4 | 4 | 3 | 3 | 3 |
| 4 | 5 | 5 | 5 | 5 | 5 | 5 | 5 | 5 | 5 |
| 5 | 5 | 4 | 1 | 4 | 2 | 4 | 5 | 3 | 3 |
| 6 | 4 | 4 | 2 | 2 | 5 | 4 | 3 | 3 | 2 |
| 7 | 4 | 4 | 1 | 4 | 2 | 2 | 1 | 2 | 2 |
| 8 | 5 | 4 | 4 | 4 | 3 | 4 | 3 | 3 | 4 |
| 9 | 2 | 2 | 4 | 5 | 5 | 4 | 3 | 3 | 3 |
| 10 | 4 | 3 | 2 | 1 | 2 | 1 | 1 | 3 | 3 |
| 11 | 4 | 4 | 3 | 3 | 4 | 4 | 3 | 2 | 4 |
| 12 | 3 | 2 | 2 | 3 | 2 | 1 | 3 | 2 | 3 |
| 13 | 4 | 3 | 3 | 4 | 4 | 5 | 4 | 2 | 3 |
| 14 | 5 | 1 | 3 | 3 | 4 | 2 | 2 | 3 | 1 |
| 15 | 2 | 2 | 1 | 1 | 2 | 1 | 3 | 1 | 3 |
| 16 | 2 | 2 | 3 | 3 | 4 | 3 | 2 | 2 | 3 |
| 17 | 3 | 4 | 4 | 4 | 2 | 2 | 2 | 3 | 3 |
| 18 | 5 | 5 | 4 | 4 | 5 | 4 | 3 | 3 | 4 |
| 19 | 4 | 4 | 3 | 4 | 4 | 4 | 4 | 4 | 4 |
| 20 | 5 | 3 | 3 | 3 | 4 | 5 | 4 | 3 | 4 |
| 21 | 3 | 2 | 3 | 3 | 3 | 4 | 3 | 4 | 3 |
| 22 | 4 | 2 | 5 | 4 | 3 | 2 | 5 | 4 | 4 |
| 23 | 3 | 3 | 5 | 5 | 5 | 4 | 4 | 5 | 4 |
| 24 | 2 | 4 | 3 | 5 | 4 | 4 | 3 | 3 | 4 |
| 25 | 3 | 2 | 2 | 3 | 2 | 3 | 2 | 3 | 2 |
| 26 | 3 | 5 | 3 | 4 | 4 | 5 | 3 | 4 | 2 |

| 27 | 3 | 2 | 4 | 5 | 3 | 2 | 1 | 1 | 3 |
| --- | --- | --- | --- | --- | --- | --- | --- | --- | --- |
| 28 | 3 | 5 | 1 | 5 | 5 | 5 | 4 | 5 | 5 |
| 29 | 3 | 4 | 4 | 4 | 5 | 4 | 2 | 4 | 4 |
| 30 | 4 | 4 | 3 | 3 | 5 | 5 | 4 | 4 | 5 |
| 31 | 3 | 1 | 2 | 1 | 3 | 3 | 3 | 1 | 2 |
| 32 | 3 | 3 | 3 | 3 | 5 | 5 | 3 | 4 | 5 |
| 33 | 1 | 2 | 5 | 5 | 3 | 5 | 1 | 5 | 4 |
| 34 | 4 | 4 | 2 | 3 | 3 | 3 | 3 | 2 | 3 |
| 35 | 3 | 4 | 3 | 3 | 4 | 4 | 4 | 4 | 3 |
| 36 | 3 | 4 | 4 | 3 | 5 | 5 | 5 | 3 | 4 |
| 37 | 5 | 4 | 3 | 3 | 1 | 3 | 1 | 5 | 2 |
| 38 | 5 | 3 | 3 | 3 | 4 | 5 | 5 | 2 | 3 |
| 39 | 1 | 3 | 1 | 3 | 4 | 4 | 3 | 5 | 4 |
| 40 | 2 | 1 | 1 | 1 | 3 | 2 | 3 | 5 | 2 |
| 41 | 2 | 2 | 4 | 3 | 2 | 2 | 2 | 4 | 3 |
| 42 | 4 | 3 | 3 | 4 | 5 | 3 | 3 | 5 | 4 |
| 43 | 3 | 3 | 1 | 1 | 3 | 3 | 2 | 3 | 2 |
| 44 | 2 | 2 | 2 | 3 | 2 | 4 | 2 | 1 | 2 |
| 45 | 2 | 3 | 5 | 4 | 5 | 5 | 1 | 1 | 3 |
| 46 | 4 | 5 | 4 | 4 | 5 | 5 | 5 | 5 | 5 |
| 47 | 3 | 2 | 2 | 2 | 3 | 4 | 3 | 3 | 3 |
| 48 | 2 | 4 | 1 | 2 | 3 | 4 | 4 | 5 | 3 |
| 49 | 2 | 4 | 1 | 2 | 5 | 3 | 4 | 2 | 3 |
| 50 | 3 | 3 | 5 | 2 | 4 | 4 | 3 | 2 | 3 |
| 51 | 2 | 2 | 4 | 2 | 3 | 3 | 3 | 2 | 5 |
| 52 | 4 | 2 | 2 | 2 | 1 | 2 | 1 | 1 | 2 |
| 53 | 3 | 3 | 2 | 2 | 4 | 4 | 3 | 3 | 2 |
| 54 | 4 | 4 | 3 | 4 | 3 | 3 | 2 | 2 | 2 |
| 55 | 3 | 4 | 4 | 4 | 5 | 4 | 4 | 4 | 3 |
| 56 | 4 | 4 | 3 | 4 | 3 | 3 | 3 | 3 | 3 |
| 57 | 1 | 1 | 3 | 3 | 5 | 5 | 4 | 4 | 1 |
| 58 | 2 | 4 | 5 | 5 | 3 | 3 | 3 | 3 | 3 |
| 59 | 4 | 2 | 3 | 2 | 3 | 3 | 3 | 4 | 5 |

| 60 | 5 | 3 | 4 | 3 | 5 | 5 | 4 | 4 | 4 |
| --- | --- | --- | --- | --- | --- | --- | --- | --- | --- |
| 61 | 2 | 5 | 4 | 1 | 4 | 5 | 1 | 3 | 1 |
| 62 | 3 | 4 | 3 | 3 | 3 | 2 | 2 | 2 | 2 |
| 63 | 2 | 3 | 5 | 3 | 4 | 4 | 3 | 1 | 4 |
| 64 | 5 | 3 | 3 | 5 | 4 | 4 | 4 | 4 | 5 |
| 65 | 3 | 3 | 4 | 3 | 4 | 1 | 4 | 5 | 3 |
| 66 | 1 | 5 | 2 | 5 | 5 | 3 | 4 | 4 | 5 |
| 67 | 4 | 5 | 4 | 4 | 5 | 4 | 4 | 5 | 3 |
| 68 | 3 | 4 | 3 | 3 | 5 | 5 | 4 | 4 | 4 |
| 69 | 1 | 1 | 2 | 1 | 3 | 4 | 1 | 2 | 2 |
| 70 | 5 | 5 | 5 | 5 | 3 | 5 | 5 | 5 | 5 |
| 71 | 1 | 2 | 4 | 2 | 3 | 3 | 2 | 2 | 3 |
| 72 | 5 | 5 | 1 | 1 | 3 | 5 | 5 | 5 | 1 |
| 73 | 4 | 2 | 5 | 5 | 5 | 4 | 3 | 3 | 4 |
| 74 | 4 | 1 | 3 | 1 | 5 | 5 | 2 | 4 | 5 |
| 75 | 1 | 5 | 1 | 1 | 1 | 2 | 4 | 3 | 3 |
| 76 | 2 | 2 | 3 | 2 | 5 | 5 | 4 | 4 | 5 |
| 77 | 4 | 5 | 5 | 5 | 5 | 3 | 3 | 1 | 2 |
| 78 | 4 | 4 | 4 | 4 | 4 | 3 | 3 | 4 | 4 |
| 79 | 5 | 5 | 4 | 1 | 2 | 3 | 4 | 4 | 5 |
| 80 | 4 | 5 | 4 | 4 | 5 | 5 | 5 | 5 | 1 |
| 81 | 2 | 2 | 3 | 5 | 5 | 4 | 3 | 3 | 3 |
| 82 | 3 | 3 | 3 | 3 | 3 | 4 | 3 | 4 | 3 |
| 83 | 3 | 3 | 4 | 3 | 4 | 5 | 3 | 4 | 3 |
| 84 | 2 | 2 | 3 | 2 | 4 | 3 | 3 | 2 | 2 |
| 85 | 4 | 5 | 3 | 3 | 3 | 3 | 4 | 2 | 2 |
| 86 | 4 | 3 | 4 | 4 | 3 | 4 | 4 | 2 | 3 |
| 87 | 2 | 2 | 4 | 4 | 5 | 3 | 1 | 3 | 3 |
| 88 | 4 | 5 | 5 | 5 | 5 | 5 | 3 | 4 | 3 |
| 89 | 1 | 4 | 1 | 1 | 5 | 5 | 5 | 5 | 3 |

| **on a 5‐point liking scale from 1=disgusting to 5=delicious** | | | | |
| --- | --- | --- | --- | --- |
| Non‐ nutritively sweetened | Sugar‐  sweetened | Non‐ nutritively sweetened | Sugar‐  sweetened | Non‐ nutritively sweetened |
| Non‐ commercial mango | Spa forest fruits | Spa forest fruits | Spa apple/cherry | Spa apple/cherry |
| 3 | 4 | 5 | 2 | 3 |
| 3 | 3 | 4 | 4 | 2 |
| 4 | 3 | 3 | 2 | 3 |
| 5 | 5 | 5 | 5 | 5 |
| 2 | 3 | 2 | 3 | 1 |
| 3 | 4 | 4 | 3 | 3 |
| 3 | 3 | 2 | 4 | 4 |
| 3 | 4 | 4 | 4 | 4 |
| 5 | 4 | 5 | 4 | 5 |
| 3 | 1 | 1 | 2 | 3 |
| 3 | 2 | 3 | 2 | 2 |
| 3 | 3 | 4 | 3 | 2 |
| 2 | 5 | 3 | 4 | 4 |
| 2 | 3 | 4 | 3 | 3 |
| 4 | 1 | 3 | 1 | 1 |
| 3 | 3 | 4 | 3 | 3 |
| 3 | 3 | 2 | 4 | 3 |
| 3 | 5 | 5 | 4 | 5 |
| 4 | 3 | 4 | 4 | 4 |
| 3 | 4 | 5 | 4 | 4 |
| 3 | 4 | 2 | 3 | 5 |
| 3 | 5 | 5 | 2 | 4 |
| 1 | 3 | 3 | 5 | 4 |
| 3 | 4 | 5 | 4 | 5 |
| 4 | 3 | 4 | 3 | 3 |
| 3 | 5 | 1 | 3 | 5 |

| 2 | 3 | 1 | 4 | 4 |
| --- | --- | --- | --- | --- |
| 5 | 5 | 5 | 3 | 5 |
| 3 | 3 | 4 | 2 | 3 |
| 4 | 3 | 5 | 4 | 4 |
| 1 | 4 | 3 | 4 | 3 |
| 5 | 4 | 5 | 4 | 4 |
| 4 | 3 | 3 | 4 | 3 |
| 3 | 4 | 5 | 4 | 2 |
| 4 | 5 | 5 | 4 | 4 |
| 3 | 4 | 5 | 5 | 4 |
| 1 | 5 | 2 | 4 | 5 |
| 4 | 4 | 5 | 5 | 3 |
| 5 | 4 | 4 | 5 | 5 |
| 3 | 4 | 4 | 2 | 5 |
| 3 | 3 | 4 | 5 | 4 |
| 4 | 3 | 4 | 2 | 3 |
| 2 | 3 | 3 | 3 | 2 |
| 1 | 5 | 5 | 3 | 4 |
| 3 | 5 | 5 | 5 | 5 |
| 3 | 5 | 3 | 5 | 5 |
| 2 | 4 | 4 | 3 | 4 |
| 4 | 3 | 5 | 4 | 3 |
| 4 | 5 | 4 | 3 | 3 |
| 3 | 5 | 4 | 3 | 2 |
| 3 | 4 | 3 | 3 | 3 |
| 4 | 5 | 3 | 5 | 4 |
| 3 | 5 | 4 | 4 | 4 |
| 3 | 4 | 2 | 1 | 1 |
| 3 | 4 | 4 | 4 | 5 |
| 3 | 4 | 5 | 4 | 3 |
| 5 | 2 | 1 | 1 | 1 |
| 4 | 4 | 5 | 3 | 5 |
| 4 | 4 | 5 | 5 | 3 |

| 3 | 5 | 4 | 5 | 2 |
| --- | --- | --- | --- | --- |
| 3 | 5 | 2 | 3 | 2 |
| 2 | 5 | 3 | 5 | 4 |
| 1 | 5 | 3 | 3 | 1 |
| 3 | 3 | 4 | 4 | 3 |
| 2 | 4 | 4 | 4 | 4 |
| 5 | 5 | 5 | 5 | 5 |
| 5 | 4 | 4 | 5 | 4 |
| 5 | 5 | 4 | 4 | 4 |
| 3 | 4 | 3 | 4 | 4 |
| 5 | 5 | 5 | 5 | 5 |
| 3 | 3 | 4 | 2 | 3 |
| 1 | 5 | 5 | 5 | 4 |
| 5 | 3 | 3 | 3 | 4 |
| 3 | 5 | 4 | 3 | 5 |
| 3 | 3 | 4 | 4 | 3 |
| 1 | 3 | 1 | 2 | 3 |
| 1 | 2 | 2 | 4 | 1 |
| 3 | 5 | 3 | 4 | 3 |
| 3 | 2 | 4 | 4 | 3 |
| 5 | 2 | 3 | 3 | 2 |
| 3 | 4 | 4 | 4 | 3 |
| 4 | 4 | 5 | 4 | 4 |
| 4 | 4 | 5 | 4 | 4 |
| 2 | 3 | 4 | 4 | 4 |
| 4 | 3 | 1 | 4 | 4 |
| 3 | 3 | 4 | 3 | 3 |
| 4 | 4 | 1 | 5 | 5 |
| 1 | 3 | 5 | 5 | 5 |
| 4 | 5 | 1 | 3 | 2 |
